# A Comparative Evaluation of Suburethral and Transobturator Sling in 209 Cases with Stress Urinary Incontinence in 8 years

**DOI:** 10.4103/0974-1216.71615

**Published:** 2009

**Authors:** Prakash Trivedi, Sylvia D’Costa, Preeti Shirkande, Shilpi Kumar, Mangala Patil

**Affiliations:** Professor and Head of Obstetrics and Gynecology Department, Rajawadi Hospital and D Y Patil Medical College, Mumbai, India; 1 Consultant Gynecologist, NILES and Aakar IVF Centre and Holy Family Hospital, Mumbai, India; 2 Clinical Assistant, NILES and Aakar IVF Centre, Mumbai, India; 3 ICOG FOGSI Certificate Trainee, NILES and Aakar IVF Centre, Mumbai, India

**Keywords:** Monarc, stress urinary incontinence slings, transvaginal tape, trivedi obturator, trivedi’s stress urinary incontinence tape, TVT-O

## Abstract

**Aims and Objectives:**

To evaluate the outcome of suburethral and transobturator sling in treatment of female stress urinary incontinence in 209 cases from 2002 to 2010. The criteria evaluated were success, failure, complications, operating time, ease of the procedure, availability and cost effectivity of the sling.

**Design and Setting:**

A retrospective comparative study was carried out at a tertiary referral centre for female urinary incontinence.

**Material and Methods:**

A total of 209 patients (females from 27 to 79 years of age) with proven stress urinary incontinence were treated by suburethral transvaginal tape (TVT) type of slings in 101 cases and transobturator Monarc type of sling in 108 cases at the National Institute of Endoscopic Surgery and Urinary Incontinence Center, Mumbai, India, from March 2002 to June 2010. The maximum follow up was for 8 years.

**Results:**

The TVT type of slings had higher complication rate like needle entering the bladder, retention of urine necessitating to cut the tape in the center and had a success rate of 94.5% compared to Monarc/Trivedi obturator tape (TrOT) type of sling with outside-in technique, which had a negligible complication (less than 1%), pain in groin or leg movement that reduced in 6 weeks and a success rate of 95%. Specially, the Indian design Trivedi’s stress urinary incontinence tape (TSUIT) and TrOT with reusable needles, the cost was only 15–20% of the international brands.

## INTRODUCTION

In India, more than 16 million women have urinary incontinence and more than 10 million women need surgery for stress urinary incontinence (SUI). There are more than 150 surgical procedures for SUI but slings have come to stay with better results, especially in the hand of experts, where it takes less time and complications. In 1996, Ulf Ulmstein introduced the tension-free transvaginal tape (TVT),[1] while De Lorme introduced transobturator sling in 2001.[2] In India, Trivedi’s stress urinary incontinence tape (TSUIT) and Trivedi obturator Tape (TrOT) were introduced in 2003 and 2007, respectively, as a cost-effective alternative.

Incidentally, more than 40% of the cases have bladder irritability, urge incontinence and frequency, corrected by tablets needing no surgery.

## MATERIAL AND METHODS

**Figure 1:**
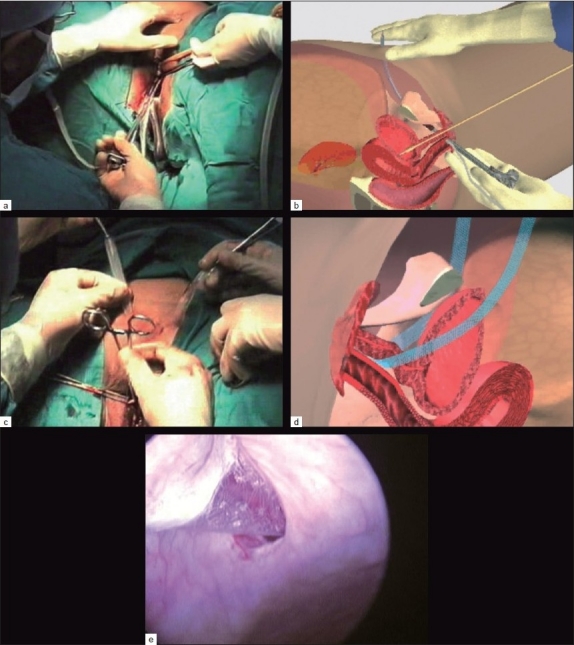
(a) TVT needle insertion on left Side with catheter and metal guide on same side, (b) Schematic insertion of TVT tape, (c) Adjusting tension free TVT, (d) Mechanism of action of mid Urethral application of TVT, (e) Bladder perforation of TVT

A total of 209 patients (females from 27 to 79 years of age) with mild to severe SUI were treated by suburethral TVT type of slings [Figure 1a–d] and transobturator Monarc type of slings at National Institute of Endoscopic surgery and Urinary Incontinence Centre, Mumbai, India, from March 2002 to June 2010.

**Figure 2:**
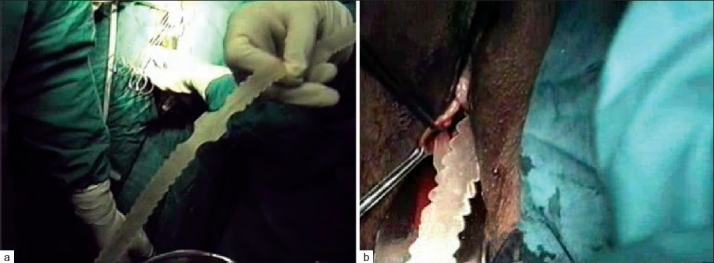
(a) Stratassis Tape (b) Stratassis tape placement

In the suburethral group, 101 cases were treated, including TVT in 7 cases, stratassis in 4 cases [Figure 2a and b], TSUIT in 90 cases, compared with transobturator technique in 108 cases predominantly outside-in techniques. We had 14 Monarc, 1 Dynamesh, and TrOT sling in 93 cases designed with reusable needle. The maximum follow up was for 8 years.

SUI diagnosis was made on a good history and clinical examination with vaginal sonography. Urodynamic study was done in only 22% of cases, since in others Q tip test and clinical history was classical & many could not afford urodynamic study.

Urodynamic study was done to confirm or rule out intrinsic sphincter deficiency (ISD) or mixed incontinence, compared to SUI, as ISD definitely needed a sling procedure. Involuntary loss of small quantity of urine due to cough, sneeze, laugh or change in position suggested GSUI excluding urgency, urge incontinence, irritable bladder or neurogenic bladder and many mixed groups which were corrected first with medicines before SUI surgery.

### Techniques

*TSUIT*: With the patient in lithotomy position under anesthesia, first vaginal dissection was done 1.5 cm from external urethral meatus, separating vagina from bladder-urethra after infiltration of 20–40 ml normal saline–0.5% sensorcaine with two drops of adrenaline. Suprapubically, 3 cm lateral from the midline on each side, 7–8 ml of 0.25% sensorcaine was injected. TSUIT needles were introduced from the dissected vagina, to come out of abdomen through a 4-mm incision. Cystoscopy with a 70° cystoscope was done to see if the needle was in bladder or very close to the bladder muscle.

In TSUIT, the tape was pulled through the abdominal wall after the TSUIT tape threads were loaded in the eye of one needle and knots were tied on both sides. After the tapes were out on both sides of the abdomen, a long artery was kept at 45° above the tape and below the urethra to make it tension-free. The procedure is easy and safe.

**Figure 3:**
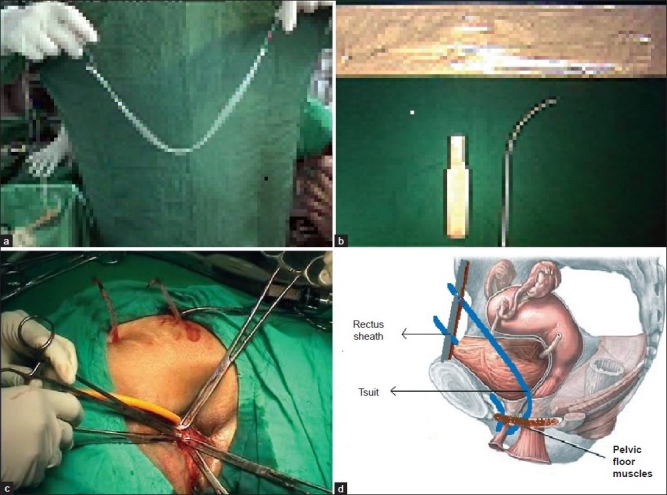
(a) TSUIT tape, (b) TSUIT handle and 4mm needle, (c) TSUIT tapes pulled out on both sides, (d) Schematic placement of TSUIT

TSUIT with reusable needle and tape [Figure 3a–d] costs Rs. 6500/- and the tape costs Rs. 1200/- only compared to international brands costing from Rs. 12,500 to 25,000/-.

Then, after Delorme’s experience, the transobturator sling got popular with no catheter, no cystoscopy required and yet comparable results.

We dislike the complicated inside-out technique of TVT-O in comparison to Monarc or TrOT or Dynamesh, which are the outside-in techniques, and are easier, successful and with minimal complications.

**Figure 4:**
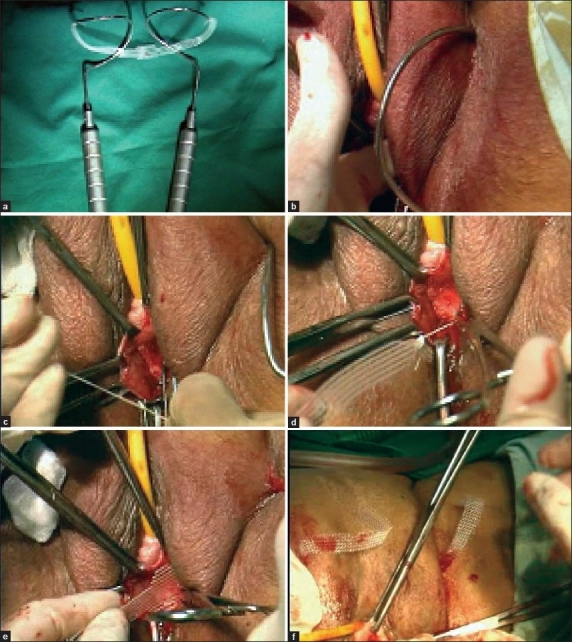
(a) TrOT tape and Needles (b) Left Needle insertion at upper and medialmost area of obturator foramen (c) Needle seen, tying threads of tape in TrOT needle’s eye (d) Pulling of TrOT tape (e) Placement of TrOT sub urethrally (f) TrOT pulled out on both sides

**Figure 5:**
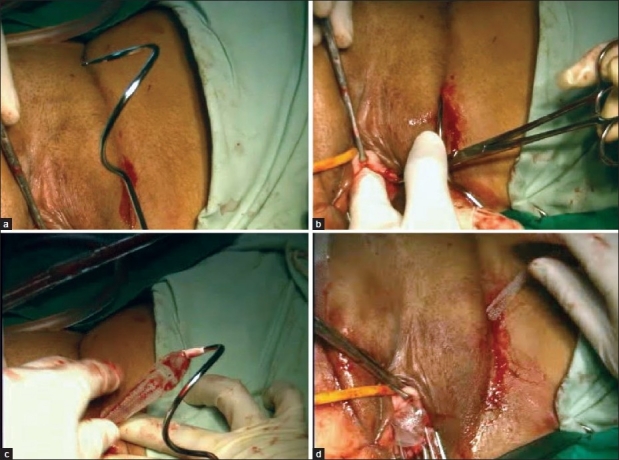
(a) Monarc needle (b) Monarc needle insertion (c) Monarc tape pulled out with Needle (d) Monarc tape placement

In TrOT [Figure 4a–f] or Monarc [Figure 5a–d] sling, the vaginal dissection was the same, the needles on each side were introduced in the genitofemoral crease at the medial most point of obturator foramen usually 2 cm above the external urethral meatus, from a 4-mm incision. The needle was first inserted perpendicularly perforating the obturator membrane. Then the shaft of the needle was turned outward by 45° and the tip of the needle was brought by rotatary movement under the inferior pubic ramus, to come out below the dissected vagina palpated by the tip of the other index finger. Then, the thread of the TSUIT tape was inserted in the eye of needle and tied in TrOT or snapped in Monarc. Needle was pulled out in anticlockwise direction. As the tape came out from both sides, a long artery at 45° was kept above the tape to avoid tension on the urethra. The excess tape projecting out of obturator foramen was cut and the vagina closed. No cystoscopy or catheter was needed.

## RESULTS

In TVT, three of seven cases were totally successful, needle or tape entered the bladder in two of seven cases [Figure 1e], and retention needing to cut the TVT tape occurred in two of seven cases with 56% complication rate. Stratassis with biodegradable material had 50% reoperation and replacement with another type of sling and was discontinued later due to intense tissue reaction.

TSUIT, used in 90 cases, had a 4-mm needle entering the bladder on both sides in 1 case of cystocele and SUI, and 5 cases of unilateral entry of the needle in the bladder. In all the cases, removal and repositioning of the needle was possible without any consequence, except that the urinary catheter was kept for 2–3 days.

**Figure 6:**
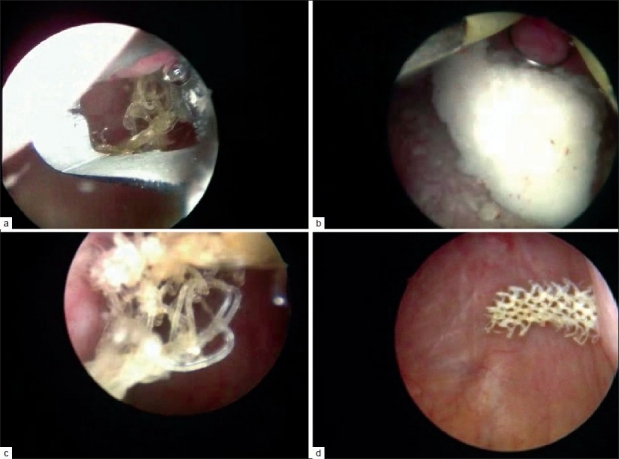
(a) Calcified tape in bladder 6 years later (b) Loupe resection of calcified tape (c) Calcified tape being removed (d) Last part of Tape in bladder

Retention needing to cut the TSUIT occurred in 2 of the 90 cases. But the general success in relief was >94.5% in TSUIT. Prolapse or herniation of tape with relief of SUI was seen in two cases, more because of poor vaginal closure needing to cut the tape and suturing the separated vagina, but SUI was totally relieved. As a late complication in one case of TVT/TSUIT, the patient came 6 years later with calcified shadows in bladder on both sides. Cystoscopy revealed calcified area on both sides with the tape going through both the walls of bladder. With the Resectoscope Loupe and Collin’s right angled electrode, tape with calcification was removed [Figure 6a–d] as much possible in the first sitting. She was relieved of SUI for the last 6 years and had no complaints. A follow-up sonography showed no remnant tape or calcified areas in the bladder. TSUIT failures in 6 years of follow up were 4 in 90 cases, i.e., <5%. Complication rate was 7%.

Stratassis in 1996 had intense tissue reaction, repeat surgery with painful nodule formation and was later discontinued.

In the group of transobturator sling after TVT-O, Monarc etc., we had 14 cases of Monarc, 1 Dynamesh and TrOT-design of outside-in technique with reusable needles in 93 cases. We also had two cases of TVT-O “inside-out” technique and were totally uncomfortable as unnecessarily surgery was made difficult compared to TrOT/Monarc etc. which are simple and easy to do.

In >80% of the transobturator tape technique, there was no need of catheterization and cystoscopy, the incidence of retention or needle entering the bladder was absent. We had one accidental bladder injury during dissection in a case of previous vaginal hysterectomy with AP repair done. Bladder was closed in layers and the obturator sling was put meticulously.

Out of 108 cases, there were 4 failures due to low placement of sling, 1 with Dynameh and 3 with TrOT.

In a redo procedure post sub urethral sling or trans obturator sling, changing to the other route i.e. transobturator or suburethral sling respectively, had > 95% success suggesting the angle of placement of sling was more flat with TrOT then TSUIT which was angled like hammock. Failure rate was 4% and complication rate was <1%.

With the TVT type of suburethral sling, TSUIT had 94% success; Monarc or similar Indian sling, TrOT, had 93.9% success and less complication rates of retention. Need of cutting the tape was higher with TVT/suburethral sling. Hence, the obturator sling is here to stay with no catheter, no cystoscopy, insignificant retention with good success and acceptable failure.

The time taken for TVT–TSUIT sling was between 30 and 60 min with an average time of 42 min, compared to transobturator sling, TrOT/Monarc which took 16–41 min with an average time of 25 min.

Both however gave >93% success rate compared to laparoscopic or open Burch which was about 70% and not advisable for ISD. The inside-out technique TVT-O was more traumatic.

Between TVT and TSUIT or TVT-O and TrOT, both TSUIT and TrOT had same results with reusable needle reducing the cost, and further, later only the tape needs to be purchased.

Dr. Barbar[3] reviewed safety in 205 patients of TOT and 213 patients of TVT at the Cleveland clinic from January 03 to August 05. The most significant difference in perioperative complication was bladder injury which occurred in 5.1% of women undergoing TVT and in no patient undergoing TOT. Voiding dysfunction was present in 8.9% of women following TVT surgery and 2.9% of TOT following 707 procedures. Anticholinergic medicines were required for 6 weeks post surgery in 14% of women in the TVT group and 6.3% in the TOT group.

Randomized trial with 3 years of follow up of TVT and TVT-O conducted at Seven Finnish hospital,[4] with 267 patients of whom 96% were evaluated; objective cure was achieved in 94.6% with TVT and 89.5% TVT-O (P-131), which was subjective. No differences in results and complication rate were found.

## DISCUSSION

Since urinary incontinence is a huge problem and GSUI/ISD needs surgery, an ideal simple cost-effective method is needed. After mastering Laparoscopic Burch, performed by us in 60 cases during 1997 to 2002, we used the new sling which brought greater promise. Further to perform laparoscopic Burch was not possible by all gynecologists and urologists.

The biggest problem with SUI surgeries is rapid change in technique not allowing proper evaluation of one method. Urodynamic study was not compulsory for all patients, except cases of failure, suspected ISD, mixed incontinence. A good history, clinical examination, and a TransVaginal Sonography (TVS) studying cough-induced urethral hypermobility were enough to diagnose GSUI and differentiate it from others needing tablets only.

We evaluated TVT, Stratassis, TSUIT, Monarc, TrOT and TVT-O in 209 patients over 8 years. The transobturator “outside-in” techniques like Monarc, TrOT, Dynamesh at this moment of time appear to be easy, safe, and successful and if the Indian modification is used, it is very cost effective. The “inside-out” technique, i.e., TVT-O had good success but higher incidence of groin or leg pain. The original TVT/TSUIT techniques are still occasionally used if the transobturator technique fails. The success is mainly because of the acute angle and hammock effect of TVT/TSUIT. Surprisingly, in patients in whom TVT or TSUIT has failed, the TOT is found to be effective. Five TOT failures treated by TVT, all having ISD,[5] were corrected with success.

Due to the proximity of TVT sling to the bladder, the sling may actually penetrate or irritate the bladder detrusor muscle, causing detrusor irritability which in some cases needs to be treated post-operatively with tablets for 4–6 weeks. TOT sling is subfascial, avoiding the retropubic space and lies in a hammock-type position under urethra, which mimics the pubourethral ligaments at less of an acute angle compared to TVT sling. This may be the reason behind the lower incidence of obstruction and voiding dysfunction in this approach.

Groin pain following the passage of needles and mesh through the TOT space and the medial groin beneath the Adductor Longus tendon is clinically significant. The inside-out approach utilized by the TVT-O studies has shown the risk of post-operative groin pain in the range of 15–24%, with as many as 4.7% of patients complaining of long-term pain.[4,5]

The risk of groin pain has been shown to be much lower in outside-in approach (including Monarc, TrOT).[6,7] For outside-in approach, the incision in the groin is made in the genitofemoral crease, 3 cm away from the obturator canal; the needle is passed through this incision directed away from the canal and the neurovascular bundle.

With a vagina–to-groin approach such as TVT-O sling, the needle is directed from the vagina out toward the obturator canal and the neurovascular bundle without finger guidance. Given the angle of the pubic bone, with this type of approach, the exit point of the needle in the groin is much more lateral to the genitofemoral crease and closer to the obturator canal compared to the Monarc/TrOT needle entry point, which may increase the risk for injury to nerves or vessels that traverse the obturator canal. The trajectory of needles and their entrance or exit points and how this may affect safety have been further discussed in many studies.[8] The bottomline is that accurate midurethral placement of the sling is required for successful treatment of SUI.

Paraurethral injections of different materials are ideally useful in some patients having SUI after delivery and who desire to have more children.

## References

[1] Hemikssonl UU, Johnson P, Vathos G (1996). An Ambulatory surgical procedure under local treatment of female urinary incontinence. Int Urogynecol J Pelvic Floor Dysfunct.

[2] Delorme E (2001). Transobturator urethral suspension;mini invasive procedure in the treatment of stress incontinence in women. Prog Urol.

[3] Moore RD, Gamble K, Miklos JR (2007). Tension free vaginal tape sling for recurrent stress incontinence after Transobturator tape sling failure. Int Urogynecol J Pelvic Floor Dysfunct.

[4] Palva K, Reme K (2010). Central Hospital, Finland. Int Urogynecol J Pelvic Floor Dysfunct.

[5] Lim J, Corrush A, Carey ME (2006). Clinical and quality of the outcomes in women treated by the TVT-O procedure. BJOG.

[6] Davila GW, Johnson JD, Serels S (2006). Multicenter experience with the monarc trans obturator sling system to treat SUI. Int Urogynecol J Pelvic Floor Dysfunct.

[7] Moore RD, Miklos JR, Cervigni M (2006). Monarc trans-obturator sling: Combined analysis of one-year following in nine countries with 266 patients. Int Urogynecol J Pelvic Floor Dysfunct.

[8] Costa P, Delmas V (2004). Transobturator tape procedure-inside out or outside in: Current concepts and evidence base. Curr Opin Urol.

